# Antimicrobial stewardship in long-term care facilities in Belgium: a questionnaire-based survey of nursing homes to evaluate initiatives and future developments

**DOI:** 10.1186/s13756-016-0106-7

**Published:** 2016-03-08

**Authors:** François Kidd, Dominique Dubourg, Francis Heller, Frédéric Frippiat

**Affiliations:** Department of Internal Medicine, Jolimont Hospital, Rue Ferrer 159, 7100 Haine-St-Paul, Belgium; Observatoire Wallon de la Santé, Direction générale opérationnelle des Pouvoirs locaux, de l’Action sociale et de la Santé, Avenue Gouverneur Bovesse 100, 5100 Namur, Belgium; Consultant in the Department of Internal Medicine, Jolimont Hospital, Rue Ferrer 159, 7100 Haine-St-Paul, Belgium; Department of Infectious Diseases and General Internal Medicine, University Hospital of Liège, Avenue de L’Hòpital 1, 4000 Liège, Belgium

**Keywords:** Antibiotic prescribing, Antibiotic resistance, Antimicrobial stewardship, Nursing home, Long-term care facilities, Belgium

## Abstract

**Background:**

The use of antimicrobials is intense and often inappropriate in long-term care facilities. Antimicrobial resistance has increased in acute and chronic care facilities, including those in Belgium. Evidence is lacking concerning antimicrobial stewardship programmes in chronic care settings. The medical coordinator practicing in Belgian nursing homes is a general practitioner designated to coordinate medical activity. He is likely to be the key position for effective implementation of such programmes. The aim of this study was to evaluate past, present, and future developments of antimicrobial stewardship programmes by surveying medical coordinators working in long-term care facilities in Belgium.

**Methods:**

We conducted an online questionnaire-based survey of 327 Belgian medical coordinators.

The questionnaire was composed of 33 questions divided into four sections: characteristics of the respondents, organisational frameworks for implementation of the antimicrobial stewardship programme, tools to promote appropriate antimicrobial use and priorities of action. Questions were multiple choice, rating scale, or free text.

**Results:**

A total of 39 medical coordinators (12 %) completed the questionnaire. Past or present antimicrobial stewardship initiatives were reported by 23 % of respondents. The possibility of future developments was rated 2.7/5. The proposed key role of medical coordinators was rated <3/5 by 36 % of respondents. General practitioners, nursing staff, and hospital specialists are accepted as important roles. The use of antimicrobial guidelines was reported by only 19 % of respondents. Education was considered the cornerstone for any future developments. Specific diagnostic recommendations were considered useful, but chest x-rays were judged difficult to undertake. The top priority identified was to reduce unnecessary treatment of asymptomatic urinary infections.

**Conclusions:**

Our study shows that the implementation of an antimicrobial stewardship programme is reported only in a minority of nursing homes. The possibility of future developments is uncertain. Nevertheless, the self-selected medical coordinators who responded to the survey reported a good knowledge of this complex problem. Despite a lack of optimism, medical coordinators seem to have the appropriate competencies to play a key role in antimicrobial stewardship in the future.

**Electronic supplementary material:**

The online version of this article (doi:10.1186/s13756-016-0106-7) contains supplementary material, which is available to authorized users.

## Background

The population of Europe is aging and there is a growing need for long-term care facilities (LTCFs) [1, 2]. The use of antimicrobials is intense in these settings. A recent review estimated that 47–79 % of residents of LTCFs receive antibiotics each year [3]. The significantly high incidence rate of healthcare-associated infections in LTCFs [4] only partially explains this observation. Several reports have estimated that systemic antimicrobials are prescribed inappropriately for 25–75 % of patients in these settings [5]. This has certainly contributed to the growing problem of antimicrobial resistance in nursing homes (NHs), including the production of extended-spectrum beta-lactamases [6], carbapenemase-producing Enterobacteriaceae [7, 8], and methicillin-resistant *Staphylococcus aureus* [9]. *Clostridium difficile* infection is also a major area of concern [10]. Diagnostic uncertainty is the major factor that account for antimicrobial overuse in LTCFs [11]. These institutions face peculiar problems that often lead to misdiagnosis and trigger inappropriate antimicrobial prescriptions. High prevalence of asymptomatic colonisation in normally sterile sites [12] and atypical presentations of infection (absence of fever and typical clinical syndromes) [13] are frequently observed in elderly patients. The complexity of the antibiotic prescribing process in LTCFs also explains uncertainties. The important role of the families and the nursing staff; difficulties of inter-professional communication; decision to prescribe frequently made off-site by telephone and/or on limited laboratory and clinical data appear to account for that complexity [14]. Restrictions on the use of antibiotics may potentially impact on the epidemiological evolution of antimicrobial resistance in the future [15]. The implementation of antimicrobial stewardship programmes (ASP) in hospitals is efficient and widely recommended [16]. Guidelines for the implementation of ASPs in LTCFs are not yet available and publications on this topic are scarce [11]. Progress in the field has been limited because of several factors. The organisation of NHs varies widely between and within countries while the health status of residents of LTCFs is also very heterogeneous. Many institutions are additionally confronted with resource limitations. Management strategies have nevertheless been proposed in recently published review articles. Their authors recommend education [14, 17, 18]; diagnostic and therapeutic guidelines [14, 17, 18]; interventions to reduce unnecessary microbiological testing and reassess antibiotic therapy after a few days [18]; integrated continuous quality improvement strategies [14, 18]; and mandatory regulatory requirements [18] as key measures to be implemented. Few studies evaluated the prevailing perceptions and attitudes of key healthcare providers about ASPs implementation in LTCFs [19, 20]. Belgium has not yet defined precise regulatory requirements for antimicrobial stewardship in LTCFs. However, the legal framework has been recently changed. The requirements about related topics such as continuous quality improvement, infection control and drug stewardship were better defined [21]. This situation led us to perform an online questionnaire-based survey of key healthcare providers to evaluate past and present actions undertaken in Belgian NHs concerning ASPs to continue to identify initiatives, barriers and possibilities for future developments in this area.

## Methods

### Survey recipients

In Belgium, residents have a freely chosen general practitioner (GP) physician to care for their individual health. Each NH must appoint a medical coordinator (MC). The MC is a GP physician designated by his peers to coordinate medical activity in the NH. Recent law changes have strengthened the position of the MCs as managers of the collective aspects of residents’ health [21]. This places the MC in an ideal position to implement ASPs at the local level. For these reasons, MCs were selected as the recipients of the questionnaire in this survey.

### Questionnaire design

To design the pilot questionnaire we reviewed recently published studies and Belgian initiatives. The studies were review articles [15, 22–24], evaluations of antimicrobial use [25–27], intervention studies [11, 28], practice guidelines [12, 29] and definitions [13, 30]. The Belgian initiatives were workshops reports and recent legal prescriptions [21, 31, 32]. The pilot questionnaire was reviewed by the antimicrobial stewardship and infection control teams of the hospitals of the Jolimont Group, Public Health experts of the Belgian Scientific Institute of Public Health and of the Belgian Antibiotic Policy Coordination Committee (BAPCOC) and an expert in infectious diseases of the University of Liège. The pilot questionnaire was tested by 4 MCs of the LTCFs of the Jolimont Group and adapted according to their advice. A final questionnaire that takes about 20 min to complete was validated. The questionnaire comprises 33 questions. These questions were divided into four sections: (1) characteristics of the respondents; (2) organisational frameworks for implementation of the ASP (programme implementation, actors to implicate in the implementation); (3) tools to promote appropriate use of antimicrobials (education, guidelines for antimicrobial use, clinical diagnostic criteria, complementary investigations); and (4) causes of inappropriate antibiotherapy that are priorities for future action. Three types of questions were asked. The first type consisted of multiple-choice questions (either yes/no or a specific list of options). The second type consisted of a self-assessment rating scale where respondents were asked to rate their agreement with statements by assigning a score of 0 (“I do not agree at all”) to 5 (“I totally agree”). The third type of question consisted of open questions where respondents were invited to provide free text comments in answer to questions. The questionnaire is available as an additional file (Additional file 1).

### Implementation of the survey

The implementation of the survey did not raise ethical issues requiring approval by an appropriate ethics committee. The survey was anonymous and without financial compensation. Participants were asked about their opinions, professional characteristics and practices. No confidential data were collected. The questionnaire was digitally encoded in French and Dutch as a Google Docs® form. In the absence of any official database of MCs in Belgium, the target sample for the survey was all the members of the French and Dutch speaking MC professional associations (*Association francophone des médecins coordinateurs* (AFRAMECO) and *Werkgroep Crataegus*). These associations forwarded the Internet link to the questionnaire and promotion emails electronically to their mailing lists. The questionnaire was sent to 327 members of the mailing lists. The main investigator of the study actively promoted the survey during a training session to MCs organised by the AFRAMECO the 15^th^ of March 2014. One reminder was emailed to all members of the mailing lists. Answers were collected from 30 April 2014 to 16 June 2014 via a Google Drive® module.

## Results

### Characteristics of the respondents

Thirty-nine (12 %) of 327 MCs participated in the survey. The multiple-choice questions (first type) and the open questions (third type) were completed to a variable degree (ranging from 26 % (10/39) to 74 % (29/39) of respondents). The rating scale questions (second type) were completed by all respondents with the exception of two questions that each received 38 responses. Among the three different regions of Belgium, the respondents were mainly working in Wallonia (77 %, 30/39), followed by Flanders (15 %, 6/39) and Brussels (8 %, 3/39). Most respondents worked in “medium size” (50–150 beds) institutions (72 %) and had been employed for more than 10 years (68 %).

### Organisational frameworks for implementation of the AMS programme

Past or present implementation of an ASP was reported in 5/22 NHs (23 %). As shown in Table 1, the majority considered that ASP could be implemented or improved in their institution. However, 14/39 (36 %) respondents indicated a score of <3/5 to this proposal. The estimated importance of ASP implementation was relatively similar for the different proposed actors (Table 1). The highest score was given to the nursing team and the lowest to the Circle of GPs. The question regarding the importance of the MCs received a mean score of 3.1/5, but this score was <3/5 for 14/39 (36 %) of respondents. In the answers to open questions, the respondents identified a number of barriers to future implementation of an ASP. Many of the respondents considered that several factors tended to lead GPs to over-diagnose and over-treat suspected infections; this includes work overload, pressure from the nursing team and/or the families of patients and fear of medical errors. According to some respondents, GPs are often opposed to external controls such as antimicrobial stewardship and infection control measures because they perceive them as limits to their freedom of prescription. They sometimes think that these measures will increase their workload. Some respondents felt that the nursing team often favoured the over-prescription of antibiotics, primarily owing to a lack of knowledge of the criteria for the appropriate use of antimicrobials. Faced with these problems, MCs often felt helpless. They also did not feel sufficiently empowered to correct their colleagues. A number of solutions to address these problems were suggested by respondents: this includes continuous education, improved communication (local consultations and contacts with colleagues, local or general media campaigns), improved collaboration (dialogue with GPs in local groups, patient-centred collaboration of different caregivers), integration in a wider context (antimicrobial stewardship in primary care) and tackling of other problems encountered in nursing homes (end of life care, nutrition, infection control, wound care).

**Table 1:** Rating scale questions about organisational frameworks to implement an antimicrobial stewardship programmes in long-term care facilities^a^

Question	Score^b^	Responses^c^	Average score
Programme implementation			
Possibility of future development in institution	0	3 (8 %)	2.7/5
	1	2 (5 %)	
	2	9 (23 %)	
	3	16 (41 %)	
	4	7 (18 %)	
	5	2 (5 %)	
Actors to involve in implementation			
Medical coordinators	0	3 (8 %)	3.1/5
	1	4 (10 %)	
	2	7 (18 %)	
	3	6 (15 %)	
	4	10 (26 %)	
	5	9 (23 %)	
Local organisation of GPs	0	6 (15 %)	2.9/5
	1	5 (13 %)	
	2	5 (13 %)	
	3	4 (10 %)	
	4	10 (26 %)	
	5	9 (23 %)	
Nursing team	0	2 ((5 %)	3.5/5
	1	4 (10 %)	
	2	2 (5 %)	
	3	9 (23 %)	
	4	9 (23 %)	
	5	13 (33 %)	
Hospital specialist (ID or AMS team)	0	2 (5 %)	3.1/5
	1	6 (15 %)	
	2	6 (15 %)	
	3	7 (18 %)	
	4	8 (21 %)	
	5	10 (26 %)	

*GP* general practitioners

*ID* infectious disease specialist

*AMS team* antimicrobial stewardship team(s) of local hospital(s)

^a^39 respondents to every question in this table

^b^Score from 0 (“I do not agree at all”) to 5 (“I totally agree”)

^c^Number of responses for each score (%)

### Tools to promote appropriate use of antimicrobials

A lack of antimicrobial guidelines in their institution was reported by 6/21 (29 %) respondents. When guidelines are present they were reported not to be in use by 11/21 (52 %) respondents. As shown in Table 2, the creation of local guidelines was not considered as a worthwhile project by 17/38 (45 %) of respondents, who gave a score <3/5. The introduction of specific training in the appropriate use of antimicrobials in NHs during basic medical studies was the most accepted proposed tool with a mean score of 4.5/5. Almost 70 % (27/39) of respondents gave a maximum score of 5/5 to the proposal of this tool (Table 2). In the open questions, respondents indicated the importance of integrating antimicrobial stewardship in the curriculum of continuous medical education (CME) of GPs. Almost all respondents (95 %, 20/21) were aware of the existence of guidelines describing specific minimal clinical criteria to start antibiotherapy in order to overcome diagnostic issues in the elderly patient. However, 12/21 (57 %) respondents reported that they do not use them in everyday practice, although they were considered useful tools to implement in the future. In the open questions, respondents felt that MCs could play an important role in creating, adapting and promoting guidelines locally. Some respondents considered that the importance of a quality first line clinical assessment of the patient by the nursing staff is underestimated. The use of complementary exams was recommended in the institutions of 13/22 (59 %) respondents. In the open questions, the respondents considered that, to be successful in the future, local guidelines concerning the use of complementary investigations must be implemented and validated by local GPs. Blood and urine samples were judged to be easy to obtain, unlike sputum and blood cultures. Some respondents considered microbiology results difficult to interpret and to correlate with other medical data. The use of chest x-rays was problematic; some respondents reported that the interpretation of x-ray results is difficult in elderly patients. The main problem is the lack of suitable local infrastructure in Belgian NHs and the need to transport patients to the hospital for radiological examinations. This was often mentioned as a major obstacle to performing an x-ray when it is indicated.

**Table 2:** Rating scale questions about tools to promote appropriate antimicrobial use in long-term care facilities

Question	Respondents^a^	Score^b^	Responses^c^	Average score
Antimicrobial guidelines				
Implementation of local guidelines is a good future project	38	0	4 (11 %)	2.9
		1	8 (21 %)	
		2	5 (13 %)	
		3	8 (21 %)	
		4	5 (13 %)	
		5	8 (21 %)	
Education				
Teaching about antimicrobial use in LTCF during medical studies	39	0	0 (0 %)	4.5
		1	1 (3 %)	
		2	0 (0 %)	
		3	3 (8 %)	
		4	8 (21 %)	
		5	27 (69 %)	
Antimicrobial stewardship training of medical coordinators	39	0	0 (0 %)	4.2
		1	1 (3 %)	
		2	1 (3 %)	
		3	6 (15 %)	
		4	13 (33 %)	
		5	18 (46 %)	
Basic training for LTCF nurses	39	0	0 (0 %)	3.7
		1	2 (5 %)	
		2	6 (15 %)	
		3	9 (23 %)	
		4	7 (18 %)	
		5	15 (38 %)	
Online continuous education	39	0	5 (13 %)	2.9
		1	4 (10 %)	
		2	5 (13 %)	
		3	9 (23 %)	
		4	7 (18 %)	
		5	9 (23 %)	
Clinical diagnostic criteria guidelines				
Useful tool for general practitioners	39	0	0 (0 %)	3.8
		1	3 (8 %)	
		2	4 (10 %)	
		3	7 (18 %)	
		4	8 (20 %)	
		5	17 (44 %)	
Useful tool for nurses	38	0	0 (0 %)	3.6
		1	3 (8 %)	
		2	8 (21 %)	
		3	6 (16 %)	
		4	6 (16 %)	
		5	15 (39 %)	
Complementary investigations guidelines				
Implementation of local guidelines is a good future project	39	0	6 (15 %)	2.9
		1	3 (8 %)	
		2	5 (13 %)	
		3	7 (18 %)	
		4	10 (26 %)	
		5	8 (20 %)	

*LCTF* long-term care facilities

^a^Number of respondents to the question

^b^Score from 0 -“I do not agree at all” to 5 -“I totally agree”-

^c^Number of responses for each score (%)

### Causes of inappropriate antibiotherapy that are priorities for future action

As shown in Table 3, respondents broadly endorsed the proposed list of priority issues, as evidenced by the average score of 3.8/5 for all questions. Respondents indicated that the highest priority is to avoid antimicrobial therapy in asymptomatic bacteriuria.

**Table 3:** Rating scale questions about priorities for future actions^a^

Question	Score^b^	Responses^c^	Average score
Treatment of asymptomatic urinary tract infections	0	0 (0 %)	4.0
	1	0 (0 %)	
	2	5 (13 %)	
	3	6 (15 %)	
	4	13 (33 %)	
	5	15 (39 %)	
Antibiotic use in viral respiratory syndromes	0	1 (3 %)	3.9
	1	1 (3 %)	
	2	1 (3 %)	
	3	8 (20 %)	
	4	16 (41 %)	
	5	12 (30 %)	
Antibiotic use in colonised chronic wounds	0	0 (0 %)	3.8
	1	1 (3 %)	
	2	4 (10 %)	
	3	6 (15 %)	
	4	17 (44 %)	
	5	11 (28 %)	
Excessive fluoroquinolone use	0	0 (0 %)	3.7
	1	3 (8 %)	
	2	3 (8 %)	
	3	7 (18 %)	
	4	13 (33 %)	
	5	13 (33 %)	
Long antibiotic durations	0	0 (0 %)	3.6
	1	2 (5 %)	
	2	4 (10 %)	
	3	9 (23 %)	
	4	15 (39 %)	
	5	9 (23 %)	

^a^39 respondents to every question in this table

^b^Score from 0 -“I do not agree at all”- to 5 -“I totally agree”-

^c^Number of responses for each score (%)

## Discussion

To the best of our knowledge, this study is the first to evaluate the implementation of ASPs in nursing homes by surveying, in a structured way, local medical professionals involved both in first line care as a GP physician and in a management position as a MC. Although precise regulatory requirements for ASPs in LTCFs are still missing in Belgium, these actors are intended by recent legislative initiatives to play a more important role in the local implementation of quality assurance programmes in Belgian NHs [21, 32]. In a recent review, Crnich et al. [14] pinpointed the importance to base the implementation of an ASPs on goal setting, process and outcome measurement, and continuous quality improvement. Dyar et al. [18] proposed to embed ASPs in existing quality/safety/infection prevention and control programmes. In our view, this supports our choice for MCs as recipients of the survey. They potentially have a key role to play in the development of the ASPs by integrating them into their quality programmes. Moreover, other data suggests that the presence of coordinating physicians in LTCFs may improve prescribing (by reducing phone prescribing), increase the use of antimicrobial guidelines and lower prevalence of antibiotic use in GP-care only LTCFs [18].

Our results show that more than two thirds of the MCs who responded, who had mostly been working in their institution for ≥10 years, were not aware of the completion of any ASP in their institution. The future implementation of a local ASP is considered feasible, but with many uncertainties. Past experiences are scant and MCs are not very optimistic. Nevertheless, they pinpointed barriers and facilitators of antimicrobial stewardship that are very similar to those previously described by Lim et al. [19] and Fleming et al. [20] in their recently published studies. According to the results of our survey, it remains controversial to place a MC in a key position for the future implementation of ASPs. Over a third of respondents rejected this proposal. The interpretation of this data is difficult but may reflect some degree of resistance to change in a number of these professionals. Moreover, only the small number of self-selected MCs who responded to the survey gave their opinion on that topic. In our opinion, it is difficult to extrapolate this result to the MCs who have not participated. To further evaluate this issue an intervention study that assesses the implementation of ASPs by MCs would be useful. Respondents often agree that it is important to provide a role to the nursing team in this field. Recently published studies also emphasise the role of nurses. It is noteworthy that Lim et al. also pinpointed nursing staff as potential facilitators of AMS initiatives due to their central position (frequent contacts with residents, families, GPs and MCs) in providing healthcare within LTCFs [19]. Dyar et al. [18] emphasised the necessity to address the nurse-physician-family triad in any future intervention because this appear to be influential in generating antibiotic prescription in chronic care settings. Even if some rejected this idea, the majority of respondents considered that hospital specialists have a role to play in the implementation of ASPs in NHs. An intervention study by Jump et al. [28] measured the impact of introducing an infectious diseases consultation service in long-term care wards. In this study, they showed a significant decrease in systemic antimicrobial use and in the incidence of *Clostridium difficile* colitis. This study was implemented in long-term care wards of acute care facilities where resident management and access to the care of a specialist differ from that of freestanding NHs, which is the case in most NHs in Belgium. Despite this, the impact of these results cannot be overlooked and this track must remain open for later developments. Future intervention studies in the European and Belgian contexts would also be helpful. The question of the extension of ASPs in hospitals to encompass LTCFs was not raised by our survey. In our opinion, many legal and psychological barriers makes it currently a difficult option to consider in Belgian settings but this path must remain open in the perspective of a greater integration of geriatric care [33]. However, the problems to be addressed in chronic care settings are very different from those encountered in acute care. In their recently published review article, Dyar et al. [18] well described the specificity of the challenges faced by ASPs in LTCFs. All this indicates that the ASPs integration problem is a complex one which requires further investigations.

The surveyed MCs generally reported accurate and adequate knowledge of the tools to promote appropriate use of antimicrobials in LTCFS. However, the use and development of antimicrobial guidelines have been greeted with a lack of enthusiasm. On the question of education, respondents are much more consensual and enthusiastic. The most acceptable proposition of this study was the introduction of a specific training programme in the appropriate use of antimicrobials in LTCFs during the basic medical curriculum. Respondents considered that this would be an early and effective training for all future GPs who would, when qualified, visit patients in NHs. Adapted and specialised education in AMS for MCs and nursing staff and CME of GPs were also judged to be cornerstones for future ASPs. Frequent situations of inappropriate use were well known by respondents. They consider that the first priority is to avoid prescribing antibiotics for asymptomatic bacteriuria, as highlighted in previous studies and reviews [22–24]. In our view, this adequate knowledge is important because, as pointed by Dyar et al. [18], it is advised to target areas where antibiotic misuse is common to implement ASPs effectively. Available guidelines about diagnostic criteria were generally known, although they were reported to be in use by less than half the respondents. They are generally considered as useful tools for the nursing team (first evaluation) and physicians. As underlined in the 2008 guidelines produced by the Infectious Diseases Society of America (IDSA) [29] accurate recognition or rejection of infectious signs is critical for treatment to be appropriate. In the United States, some LTCFs have assigned nursing staff an important role. Nurses refer to a checklist which can lead them to suggest appropriate tests and indications for antibiotic treatment before assessment by a physician who confirms or not the decision. In Belgium, such measures do not appear necessary owing to a greater availability of physicians. However, the important role of nurses in the initial management of infections is recognised by our respondents. The use of basic biological and radiological (chest x-ray) complementary examinations is recommended in the majority of the institutions. Even if an abnormal chest x-ray is considered the most reliable method of diagnosis for healthcare-associated pneumonia, the performance of this examination is rarely done in practice according to our MCs. The authors of the IDSA guidelines (2008) indicate that the current literature shows considerable variability regarding the execution of chest radiographs; the proportion of community-based nursing facilities performing chest radiographs ranged from 20 % to 35 %, reaching as high as 85 % in university medical centre affiliate nursing facilities [29]. It is important to note that those data originated from the United States where chronic care settings have often a better accessibility to technical investigations. According to our respondents, the major difficulty is linked to the absence of the radiological equipment necessary to do those tests in Belgian NHs. In addition, each radiological indication requires the patient to be transported to the hospital by ambulance, a service that is costly and resource consuming. This problem remains to be solved in Belgium as the clinical diagnosis of pneumonia can be difficult and, excluding bacteraemia from all sources, pneumonia is the leading infectious cause of mortality for residents of LTCFs [29]. Obtaining blood cultures was frequently and, to our view, inaccurately judged to be impossible. This fact may be linked to an inappropriate knowledge about current diagnostic methods of infection. Respondents did not recognise any difficulties about blood sampling and processing for basic investigations. However, they do not seem to be aware that handling of blood culture samples does not require any special procedures as they can be kept at room temperature and sent to the laboratory with other specimens. In our opinion, the implementation of standardised “infection kits”, including basic biological examinations and blood culture samples for the investigation of a suspected systemic infections, could be beneficial. Some microbiological investigations (urine cultures, wound swabs) should, by contrast, be discouraged without a reasoned clinical indication as it often serve as a trigger for unnecessary antimicrobial use [18]. Any further effort to implement effective ASPs should therefore specify precisely laboratory tests indications. The implementation of “infection kits” and guidelines for their appropriate use could warrant exploration in subsequent interventions studies.

The main limitation of our study is the limited number of respondents (12 %) leading to a significant selection bias. Several factors can explain this result. Our ability to communicate with the Belgian MCs was limited in the absence of an official and public database in Belgium. Confidentiality and data security issues prevented the professionnal associations of MCs to fully share their private databases. We sent our informations indirectly to their members. Additionnaly, temporal and financial constraints prevented the deployment of a more effective communication plan to promote our survey. The low response rate among professionals could reflect a lack of interest about the topic. It could also highlight the lack of awareness about the importance of the issue. In our opinion, even with a small sample size, the survey nevertheless responds to its main objective wich was to continue to identify initiatives, barriers and possibilities for future developments in the field. Most replies came from one region of Belgium (Wallonia). This fact could be linked to the characteristics of the investigators of the survey [34]. They were better known by some French speaking physicians and the main investigator (first author of this article) actively promoted the survey during a training session to MCs organised by the French speaking professionnal association of MCs (AFRAMECO) just before the implementation of the survey.

## Conclusion

Our study shows that initiatives concerning antimicrobial stewardship are reported by responding MCs only in a minority of LTCFs. Even if they are sometimes pessimistic before the huge task to achieve, the small sample of self selected MCs who responded to the survey seems to possess the competences required to play a key role in the local implementation of future ASPs. Several issues identified by this study (MC, GPs, nursing staff, infectious disease specialist roles and complementary exams use guidelines) should be subsequently evaluated by prospective intervention studies.

## Additional file

Additional file 1: Questionnaire sent to medical coordinators. (PDF 132 kb)
