# Supplementary material for: Antimicrobial stewardship in long-term care facilities in Belgium: a questionnaire-based survey of nursing homes to evaluate initiatives and future developments
Source: Antimicrob Resist Infect Control. 2016 Mar 8;5:7. doi: 10.1186/s13756-016-0106-7 (PMC4784372; doi:10.1186/s13756-016-0106-7)
Supplement: Additional file 1: — Questionnaire sent to medical coordinators. (PDF 132 kb) [file 13756_2016_106_MOESM1_ESM.pdf]

QUESTIONNAIRE

Antimicrobial stewardship in nursing homes

- **Who are you?**

This questionnaire is anonymous. Nevertheless, i would like to collect some general informations about you and your institution.

1. In wich province is located your institution?

- Brussels
- Vlaams Brabant
- Brabant wallon
- Antwerpen
- Liège
- Limburg
- Hainaut
- Oost Vlanderen
- Luxembourg
- West Vlanderen
- Namur

2. Number of beds in your institution ?

- <50
- 50 to 150
- >150

3. How long do you work as medical coordinator?

- less than 3 years
- 3 to 10 years
- more than 10 years

- **Implementation of an organisational framework for AMS in nursing homes.**

While preserving the therapeutic freedom of the prescribers, the question of the need for a global antimicrobial stewardship (AMS) program in the healthcare institution is asked.

.

4. In your institution, an AMS program is or was previously implemented ?

- Yes
- No
- I don't know

5. Comments.If yes could you describe it briefly ?

6. In your institution, it is possible to implement or improve an AMS program. Give a score 0 to 5

	0	1	2	3	4	5	
I do not agree at all	○	○	○	○	○	○	I totally agree

7. Comments.Don't hesistate to give a free comment about this topic.

The actors of AMS

8. The medical coordinator should play a central role in AMS by coordinating cooperation among the various actors. Score 0 to 5.

	0	1	2	3	4	5	
I do not agree at all	○	○	○	○	○	○	I totally agree

9. The Local organisation of GPs implicated in NH patient care must be implicated in the AMS program definition. Score 0 to 5

	0	1	2	3	4	5	
I do not agree at all	○	○	○	○	○	○	I totally agree

10. The nursing team, as a first line actor in resident care, can play an important role in order to help to detect infections and to manage antibiotics on a daily basis. Score 0 to 5.

	0	1	2	3	4	5	
I do not agree at all	○	○	○	○	○	○	I totally agree

11. One or more members of the AMS or the infectious diseases specialists of the hospitals functionally linked with your institution must be involved in the definition of the AMS program. Score 0 to 5.

	0	1	2	3	4	5	
I do not agree at all	○	○	○	○	○	○	I totally agree

12. Comments. You can express yourself freely about the AMS actors.

- **Tools facilitating appropriate antimicrobial use**

Antimicrobial formulary implementation

One of the elements of an effective program is the implementation and use of a locally defined antimicrobial formulary.

13. In my institution the antimicrobial formulary . ..

- is inexistant.
- consist in official recommendations but is unused.
- consist in promoted and used offical recommendations.
- consisted in promoted and used local recommendations based on official ones.

14. In my institution, the development of a concerted and promoted antimicorbial formulary could be a nice project to initiate a dynamic among prescribers. Score 0 to 5.

	0	1	2	3	4	5	
I do not agree at all	○	○	○	○	○	○	I totally agree

15. Comments about antimicrobial formulary ?

The role of education

Education is the cornerstone on wich to found quality healthcare. A more rational use of antimicrobial is an important point to teach.

16. A teaching about appropriate use of antimicrobials in the institutionnalised elderly must be included in basic medical cursus. Score 0 to 5.

	0	1	2	3	4	5	
I do not agree at all	○	○	○	○	○	○	I totally agree

17. A specific teaching to AMS must be proposed to medical coordinators in nursing homes.

	0	1	2	3	4	5	
I do not agree at all	○	○	○	○	○	○	I totally agree

18. A basic teaching about appropriate use of antimicrobials is necessary for the nursing staff of the nursing home (fhighly implicated first line healthcare workers).

	0	1	2	3	4	5	
I do not agree at all	○	○	○	○	○	○	I totally agree

19. E-learning is a solution especially for continuous education.

	0	1	2	3	4	5	
I do not agree at all	○	○	○	○	○	○	I totally agree

20. Comments

Use of Minimal clinical criteria for starting antibiotherapy to overcome diagnostic issues

The clinical diagnosis of infection can be difficult in the elderly (absence of fever, attenuated focal signs,..). Deciding when to start an empirical antibiotherapy can be challenging. Several scientific societies have proposed Minimal clinical criteria for starting antibiotherapy**.** For example, for a resident without an urinary catheter, the minimal criteria to begin an antibiotheray could be an isolated dysuria or T° > 37.9°C and at least one of the following criteria : new or worsening urgenturia/ an increasing of frequence/ macroscopic hematuria/ pain at the costovertebral angle or apparition of a new urinary incontinence (SHEA 2001). The goal of this strategy is to limit antimicrobial use to those specfific criteria.

21. This type of recommandations are …

- Known by me and my colleagues and used in my institution
- Known by me and my colleagues but were never used in my institution
- Unknown by me and my colleagues

22. Those recommandations are useful tools and promoting them among precscribers can be a nice project for my institution.

	0	1	2	3	4	5	
I do not agree at all	○	○	○	○	○	○	I totally agree

23. Those recommandations could be used by the nursing team to suggest adequate therapeutic indications based on rational criteria to the prescriber.

	0	1	2	3	4	5	
I do not agree at all	○	○	○	○	○	○	I totally agree

24. Comments :

Use of complementary investigations to overcome diagnostic issues

Beyond this first diagnostic approach, the use of simple complementary exams as often as possible (urine and sputum analysis, blood cultures, Chest X Ray) can help improve patient care and allows to know better the local epidemiology.

25. The use of those complementary examinations is recommended in your institution ?

- Yes
- No
- I don’t know

26. The future implementation of local guidelines about the use of those tools in my institution is useful.

	0	1	2	3	4	5	
I do not agree at all	○	○	○	○	○	○	I totally agree

27. Comments:

- frequent causes of inappropriate antibiotherapy which are priorities for future actions.

Current data (Belgium, Europe, America) regarding the use of antibiotics in chronic care facilities clearly demonstrate that some types of "inappropriate use" are more frequent than others. Below, the main issues are presented. I would like your feeling as field practitioner on the importance and priority to be given to either of these problems.

28. In a population of elderly institutionnalised 30 to 50% of the residents presented an asymptomatic bacteriuria. Current scientific evidence suggest that it’s linked to aging and does not contituted a significant risk factor to develop a clinical infection. However, a large proportion of the observed antibiotic prescription is intended to "treat" these asymptomatic infections (prophylaxis). This issue is a top priority for future actions.

	0	1	2	3	4	5	
I do not agree at all	○	○	○	○	○	○	I totally agree

29. Chronic wounds are fequently colonised by bacteria without clinical infection. Antibiotics are not relevant in these situations though they are frequently prescribed. This issue is a top priority for future actions.

	0	1	2	3	4	5	
I do not agree at all	○	○	○	○	○	○	I totally agree

30. Viral respiratory infections and flu syndromes are frequently « covered » with antibiotics … not active on viruses. This issue is a top priority for future actions.

	0	1	2	3	4	5	
I do not agree at all	○	○	○	○	○	○	I totally agree

31. Fluoroquinolones are frequently overused in long term care facilities but these antibiotics have more frequent side effects (neurological adverse events, severe tendinopathy, multiresistant bacteria, clostridium superinfection) in the elderly population. This issue is a top priority for future actions.

	0	1	2	3	4	5	
I do not agree at all	○	○	○	○	○	○	I totally agree

32. Moderate to severe bacterial infections such as pneumonia require no more than seven days of antibiotic therapy. Though in situations often less critical we see longer prescriptions. This issue is a top priority for future actions.

	0	1	2	3	4	5	
I do not agree at all	○	○	○	○	○	○	I totally agree

33. Comments
